# Is Fatigue a Disease?

**Published:** 1921-02-12

**Authors:** 


THE PUBLIC WELL-BEING—(*continued*).

### Is Fatigue a Disease?

As our capacity for dealing with disease increases we seem to be faced by so many new discoveries as to suggest that mankind will never be able to grapple with it on equal terms. To suggest that fatigue is a disease appears merely to be an extension of the handicap against us in our search for social health. But it is no good winning in one part of the field if we are routed in another, and the extension of the battle-line against disease by the discovery of new forms of disease, though it multiplies the tasks, gives greater opportunities for dealing with them. The most effective way of dealing with an old disease may well be to attack a new one, and there can be no doubt that fatigue adds to the doctor's problems and is a nursery for disease and a handicap in coping with it. There are, undoubtedly, some workers who are always tired, not merely in the ordinary sense of feeling disinclined for effort, but in the more serious sense, from a medical and social point of view, of being always abnormally susceptible to ailment, and always below what ought to be their normal power of production.

The manufacture during the war of enormous quantities of articles of the same kind revealed amazing differences between one worker and another, and between the same worker at different hours of the day; even though this has been scarcely touched as a subject of research, in spite of its importance, at different seasons of the year. Since the war these investigations have been continued by the Fatigue Research Board, now unhappily threatened with extinction as an economy. It is rather as if a motorist were to decide to save money by doing without maps. To the medical profession this research is of the utmost interest because it is bound to throw light on the question, scarcely ever discussed, of whether in present conditions a large part of the effort of doctors and nurses is not being thrown away in trying to keep people up to a standard of strain to which they ought never to be subjected under conditions which ought not to be allowed. It may not appear to be of immediate medical interest to be told, as we now are, by the Fatigue Research Board that the methods employed in some steelworks, while far less productive than those employed in others, also impose a far greater strain on the workers; but, if the medical men separately responsible for the health of these different sets of workmen could compare notes, they would possibly find that the workman's long hours of unproductive work are productive of long hours of work for the doctor as wasteful to society as uneconomical workshop methods. The correlation between fatigue and workshop accidents has been abundantly explained. It is uncanny to peruse these figures and read the tale of slight and serious accidents increasing in intensity through the heavy hours of the day and towards the end of the week, and lightening as the prospect of release supplies a fresh stimulus to keep the worker going.

It is clear that, if fatigue is not actually to be called a disease, it is a most active agent in producing and leading up to accidents and disease of every kind. It is not escapable, for the Old Testament simply summed up the observation of the common man in the phrase, "In the sweat of thy

**THE PUBLIC WELL-BEING**—(*continued*).

brow shalt thou eat bread"; but to reduce it to its minimum and prevent it when it is unnecessary is an interest of the doctor just as much as it is now seen to be the interest of the employer and of the society which he and his workmen together serve. How much the interests of the medical profession and those in charge of the country's production are the same is suggested by the fact that fourteen million working hours a year are lost through sickness. The study of fatigue and how it can be avoided may well provide some of the most powerful allies of the medical profession and do much to make practicable the vision of a world where we shall not merely be fighting a back-to-the-wall battle with disease, but keeping the nation up to a standard of fitness for work and play and life which at present is far beyond us.

